# Supplementary material for: Dopamine transients do not act as model-free prediction errors during associative learning
Source: Nat Commun. 2020 Jan 8;11:106. doi: 10.1038/s41467-019-13953-1 (PMC6949299; doi:10.1038/s41467-019-13953-1)
Supplement: Supplementary file 2 — Reporting Summary [file 41467_2019_13953_MOESM2_ESM.pdf]

nature research

Corresponding author(s): *Double-blind peer review submissions: write DBPR and your manuscript number here instead of author names.*

Last updated by author(s): *YYYY-MM-DD*

Reporting Summary

Nature Research wishes to improve the reproducibility of the work that we publish. This form provides structure for consistency and transparency in reporting. For further information on Nature Research policies, see Authors & Referees and the Editorial Policy Checklist.

Statistics

For all statistical analyses, confirm that the following items are present in the figure legend, table legend, main text, or Methods section.

n/a	Confirmed	
☐	☒	The exact sample size (*n*) for each experimental group/condition, given as a discrete number and unit of measurement
☐	☒	A statement on whether measurements were taken from distinct samples or whether the same sample was measured repeatedly
☐	☒	The statistical test(s) used AND whether they are one- or two-sided<br>*Only common tests should be described solely by name; describe more complex techniques in the Methods section.*
☒	☐	A description of all covariates tested
☒	☐	A description of any assumptions or corrections, such as tests of normality and adjustment for multiple comparisons
☒	☐	A full description of the statistical parameters including central tendency (e.g. means) or other basic estimates (e.g. regression coefficient) AND variation (e.g. standard deviation) or associated estimates of uncertainty (e.g. confidence intervals)
☐	☒	For null hypothesis testing, the test statistic (e.g. *F*, *t*, *r*) with confidence intervals, effect sizes, degrees of freedom and *P* value noted<br>*Give P values as exact values whenever suitable.*
☒	☐	For Bayesian analysis, information on the choice of priors and Markov chain Monte Carlo settings
☒	☐	For hierarchical and complex designs, identification of the appropriate level for tests and full reporting of outcomes
☒	☐	Estimates of effect sizes (e.g. Cohen's *d*, Pearson's *r*), indicating how they were calculated

Our web collection on statistics for biologists contains articles on many of the points above.

Software and code

Policy information about availability of computer code

Data collection	Data were collected using commercially available programs as described in the text.
Data analysis	Data were analyzed using commercially available programs as described in the text.

For manuscripts utilizing custom algorithms or software that are central to the research but not yet described in published literature, software must be made available to editors/reviewers. We strongly encourage code deposition in a community repository (e.g. GitHub). See the Nature Research guidelines for submitting code & software for further information.

Data

Policy information about availability of data

All manuscripts must include a data availability statement. This statement should provide the following information, where applicable:

- Accession codes, unique identifiers, or web links for publicly available datasets
- A list of figures that have associated raw data
- A description of any restrictions on data availability

The data that support the findings of this study, and any associated custom programs used for its acquisition, are available from the corresponding authors upon reasonable request.

Field-specific reporting

Please select the one below that is the best fit for your research. If you are not sure, read the appropriate sections before making your selection.

☐ Life sciences ☒ Behavioural & social sciences ☐ Ecological, evolutionary & environmental sciences

Behavioural & social sciences study design

All studies must disclose on these points even when the disclosure is negative.

Study description	All data collected are quantitative, with the exception of histological analyses, which is a subjective qualitative judgement of whether virus expression and fiber placement reach a pre-determined criterion.
Research sample	Our rats are taken from our breeding colony of TH-Cre rats, initially sourced from RRRC Missouri and bred with Charles River wild type rats.
Sampling strategy	All groups are matched by age and sex.
Data collection	All data are collected with automated software described in the text, with the exception of histological analyses. In the case of histological analysis, experimenters are blind to group allocations.
Timing	Data were collected across a period beginning 06/19/2017-09/30/2018.
Data exclusions	Our total N is 74, we had 18 exclusions across four experiments. 10 of these exclusions are from histological analyses, due to damage in brain tissue, misplacement of fibers, or unilateral/minimal viral expression. The remaining rats were excluded due to behavioral abnormalities or infection.
Non-participation	N/A
Randomization	All rats are randomly allocated to groups, which are matched by sex and age.

Reporting for specific materials, systems and methods

We require information from authors about some types of materials, experimental systems and methods used in many studies. Here, indicate whether each material, system or method listed is relevant to your study. If you are not sure if a list item applies to your research, read the appropriate section before selecting a response.

Materials & experimental systems

n/a	Involved in the study	
☒	☐	Antibodies
☒	☐	Eukaryotic cell lines
☒	☐	Palaeontology
☐	☒	Animals and other organisms
☒	☐	Human research participants
☒	☐	Clinical data

Methods

n/a	Involved in the study	
☒	☐	ChIP-seq
☒	☐	Flow cytometry
☒	☐	MRI-based neuroimaging

Animals and other organisms

Policy information about studies involving animals; ARRIVE guidelines recommended for reporting animal research

Laboratory animals	Long evans rats.
Wild animals	None
Field-collected samples	None
Ethics oversight	ACUC protocol approved as described in text.

Note that full information on the approval of the study protocol must also be provided in the manuscript.
